# Multi-Walled Carbon Nanotubes-Based Sensors for Strain Sensing Applications

**DOI:** 10.3390/s21041261

**Published:** 2021-02-10

**Authors:** Anindya Nag, Md. Eshrat E Alahi, Subhas Chandra Mukhopadhyay, Zhi Liu

**Affiliations:** 1 School of Information Science and Engineering, Shandong University, Jinan 251600, China; liuzhi@sdu.edu.cn; 2 The Institute of Biomedical and Health Engineering, Shenzhen Institutes of Advanced Technology, Chinese Academy of Sciences, Shenzhen 518055, China; ealahi@yahoo.com; 3 School of Engineering, Macquarie University, Sydney 2109, Australia; subhas.mukhopadhyay@mq.edu.au

**Keywords:** multi-walled, carbon nanotubes, flexible, sensors, strain

## Abstract

The use of multi-walled carbon nanotube (MWCNT)-based sensors for strain–strain applications is showcased in this paper. Extensive use of MWCNTs has been done for the fabrication and implementation of flexible sensors due to their enhanced electrical, mechanical, and thermal properties. These nanotubes have been deployed both in pure and composite forms for obtaining highly efficient sensors in terms of sensitivity, robustness, and longevity. Among the wide range of applications that MWCNTs have been exploited for, strain-sensing has been one of the most popular ones due to the high mechanical flexibility of these carbon allotropes. The MWCNT-based sensors have been able to deduce a broad spectrum of macro- and micro-scaled tensions through structural changes. This paper highlights some of the well-approved conjugations of MWCNTs with different kinds of polymers and other conductive nanomaterials to form the electrodes of the strain sensors. It also underlines some of the measures that can be taken in the future to improve the quality of these MWCNT-based sensors for strain-related applications.

## 1. Introduction

The intervention of sensors in the microelectronics world has increased the pace of life to a great extent. In earlier times, when most of the data signal collection and processing was done manually, a considerable amount of time and money was required. The initial days of sensorial prototypes saw the popularization of semiconducting sensors [1,2]. These devices were mainly developed using the microelectrochemical (MEMS) technique [3,4]. These sensors used silicon to form the substrates due to their dynamic nature in terms of tolerating harsh conditions, robustness, small size, and wide range of temperature and humidity [5]. Although these sensors served a great purpose, there were certain limitations that deterred their widespread consideration for multifunctional applications. Some of the disadvantages related to the use of MEMS-based silicon sensors can be attributed to their instability in their responses when operated over a long time period of time, high signal-to-noise ratio (SNR) at low frequencies, inability to handle high power and irregular behavior at higher frequencies [6]. Other than characteristically, these sensors have also found challenges in terms of fabrication process due to the requirement of clean-room facilities and expertise to handle the complexities in the steps of fabrication. For these reasons, sensors have been developed using materials that altered the electrical and mechanical characteristics. 

Flexible sensors have been made from different kinds of polymers and nanomaterials based on their electrical, mechanical, and thermal characteristics [7,8]. The wearable nature of the flexible sensors [9,10] has also increased their value due to their ubiquitous sensing capabilities. While considering the type of polymer to fabricate a flexible sensor, some of the primary attributes that are considered are Young’s modulus, high permeability toward heat and temperature, and ability to integrate with nanomaterials to form composites. A few of the polymers used to fabricate the flexible sensors are polydimethylsiloxane (PDMS) [11,12,13], polyethylene terephthalate (PET) [14,15,16], polyethylene naphthalate (PEN) [17,18,19], polyimide (PI) [20,21,22], polyvinyl chloride (PVC) [23,24,25], and poly(3,4-ethylenedioxythiophene) polystyrene sulfonate (PEDOT: PSS) [26,27,28]. Each of these materials has individualistic advantages and disadvantages associated with them. For example, PDMS is low cost, highly optically transparent, and biocompatible in nature, thus is favored over a wide variety of applications. However, there are certain drawbacks, like the deposition of metals and high hydrophobic nature, which has led to the use of other polymers. Although certain processes like plasma treatment can change the hydrophilicity of the PDMS surface, they will eventually revert back when in contact with air. In comparison, PET is much stronger and lighter than PDMS, which makes them perfect for barring moistures and gases. PET and PEN also exhibit high insulating properties similar to PDMS, but their higher tensile strength and stiffness makes them ideal for forming commercial sensors. The advantages of PI have been largely attributed to their high heat resistance, high wear resistance, and excellent dimensional stability. These factors make this polymer ideal for use in critical applications like aerospace aviation and forming laminating resins and high-temperature structural adhesives. PVC might not be as strong as PI in terms of mechanical tear or heat stability, but its easy welding nature has led the researchers to ingrate it with different kinds of nanomaterials for forming flexible sensors [29]. This material has been used to form sensors that are not subjected to fire as a result of the emission of toxic fumes. Polymers like PEDOT: PSS have recently been popularized due to their conductive nature, which has revolutionized the nature of composites and multi-layered structured prototypes to a great extent. However, there are still some disadvantages associated with PEDOT: PSS including its acidic nature and high stocking of insulating PSS. Similarly, different kinds of nanomaterials [30,31] have been considered to develop the electrodes of the flexible sensors. The characteristics of nanomaterials that are analyzed before choosing them for synthesis purposes are high electrical conductivity, mechanical flexibility, optical transparency, and the ability to form high interfacial bonding with the polymers. The conductive nanomaterials for flexible sensors can be broadly classified into two forms, namely carbon-based allotropes and metallic nanomaterials. These nanomaterials consist of various shapes like nanosheets [32,33], nanorods [34,35], nanoribbons [36,37], nanowires [38,39], and nanoparticles [40,41]. While the first category includes carbon nanotubes (CNTs) [42,43,44], graphene [45,46,47], and graphite [48,49,50], the second category primarily includes gold [51,52,53], silver [54,55], aluminum [56,57], and copper [58,59]. Among these conductive materials, CNTs have been used a lot due to their advantages like lightweight, small size, high specific area, high aspect ratio, and high thermal and chemical stability. Other than these attributes, their biocompatible nature has increased their usage for biomedical applications [60,61,62,63,64] to a large extent. CNTs are categorized into two classes including single-walled carbon nanotubes (SWCNTs) [65,66,67] and multi-walled carbon nanotubes (MWCNTs) [68,69,70] based on their size, structural, and electromechanical characteristics. Comparing these two types of CNTs, MWCNTs are advantageous over SWCNTs for certain reasons like low cost, ease of mass production, easy functionalization, and enhanced stabilities. As a result of this, these MWCNTs have been used for different kinds of electrochemical [71,72,73] and strain-sensing [74,75,76] applications. Out of these two genres, the use of MWCNTs to determine a spectrum of strain values has been widely done to exploit their mechanical characteristics. These nanotubes were conjugated with different kinds of nano and polymeric materials to form prototypes that were able to successfully detect the micro- and macro-natured strain values. Strain sensors have been operated for biomedical [77,78] and industrial [79,80] applications, where a certain degree of strain caused by tactile and contact forces has led to the change in the responses of the prototypes. These types of sensors have been able to perform well in research environments with controlled temperature and humidity conditions due to a couple of reasons. First, the real-time operations of the prototypes introduce interruptions in terms of unwanted vibrational motions and connection with the conditioning circuits. This disturbs the stability of the responses. Second, the deployment of the sensors is done with chosen people, specifically researchers, and for a limited amount of time whereas real-time operations of these sensors are done for a longer period of time with a larger group of people. This is why, although these sensors have functioned successfully in research laboratories, they have still not been commercialized for the market. Based on their enhanced performances, there is a need for commercialization, which would not only lead to the use of these sensors for chosen applications, but will also form a podium for future researchers to fabricate and operate novel sensors. 

Although many review articles have been published in this area [81,82,83,84,85], there are a couple of aspects that make this paper favorable and interesting for the readers. First, none of the papers as of yet have been particularly focused on the use of MWCNTs for strain-sensing applications. Although both SWCNT- and MWCNT-based strain sensors have been capable of displaying excellent performances, the morphological advantages of MWCNTs over SWCNTs have always demanded further analysis. Due to a high interfacial bonding of MWCNTs with the polymer matrix, the sensors tend to revert back to their original position, even with the presence of micro-cracks formed under the influence of applied strain. Additionally, lower cost and higher purity are the advantages of MWCNTs over SWCNTs, which has led to a shift in focus in using MWCNTs to develop flexible strain and pressure sensors. From a fabrication point of view, the bulk formation of MWCNTs is easier, along with enhanced electrical, thermal, and chemical stabilities. Even though SWCNTs provide a structural simplicity, a higher degree of functionalization and comparatively minimized tendency to form agglomerates has recently popularized the use of MWCNTs as a favorable carbon-based nanomaterial. Over a broader aspect, research on SWCNT-based sensors is still continuing equally, but mostly to develop electrochemical sensors due to their high chiral nature [86]. Second, a categorical view of the exploitation of MWCNTs for forming strain sensors is necessary to deduce the impact of processing materials on the performance of the prototypes. Most of the review articles have generalized on the employment of CNTs for strain and pressure-sensing applications, thus making it a requirement to particularly focus on MWCNT-based strain sensors. Table 1 highlights a comparative study on some of the significant works done on MWCNT-based strain sensors based on certain parameters. It can be seen that with the change in processing materials, the corresponding quality of the prototypes varied. 

The rest of the manuscript is organized as follows. Following a brief introduction given in Section 1 related to the need for MWCNT-based sensors for strain-sensing applications, the design, development, and deployment of these sensors for strain-sensing applications are explained in Section 2. This part has been sub-categorized into two sections based on the nature of the MWCNT-based sensors and these conductive allotropes were exploited in both pure and composite forms to form the resultant prototypes. Then, the future scope of these MWCNT-based sensors for strain and other similar applications is depicted in Section 3, where some of the possible measures are presented. The final section presents our conclusions of the extensive review done on this area.

## 2. Multi-Walled Carbon Nanotube (MWCNT)-Based Strain Sensors

**Figure 1:**
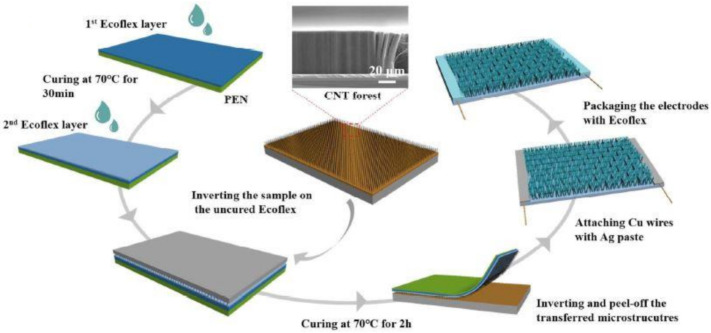
Fabrication of flexible strain sensors using carbon nanotubes (CNTs) and EcoFlex nanofin structures [97]. Reproduced from Zhang, S.; Wen, L.; Wang, H.; Zhu, K.; Zhang, M. Vertical CNT–EcoFlex nanofins for highly linear broad-range-detection wearable strain sensors. *J. Mater. Chem. C* **2018**, *6*, 5132–5139.

The utilization of MWCNTs for strain-sensing applications is highly effective due to the enhanced electrical, mechanical, and thermal qualities of the nanotubes compared to the other types of carbon allotropes. While using these MWCNTs in pure form, they primarily operate on the basis of the nature in which they are presented on the electrodes. For example, the presence of MWCNTs in different forms assists in increasing the sensitivity of the prototypes. Figure 1 [97] shows the fabrication process of flexible sensors developed from EcoFlex and vertically-aligned CNTs. These vertically aligned CNTs help in the precise measurement of the applied strain by preserving and accentuating the unique anisotropic properties of the individual nanotubes. The alignment also helps the sensors in possessing a morphology that can be easily customized and controlled to tune the sensitivity of the prototypes. The free-standing films used in the pure sensors also impart superb mechanical strength, low bulk density, and outstanding thermal conductivities. In the composite forms, the polymers provide additional attributes like biocompatibility, robustness, and overall sensitivity to the prototype. In addition to the distinctive mechanical flexibility and electrical properties exerted by the nanocomposites as a result of the tunneling effect caused by the internal conductive network, they also impart superior mechanical properties in terms of modulus and strength, noise damping, and corrosion resistance. The structural integrity of the MWCNT-based composite sensors is also much stronger, which helps in achieving long-term stability in terms of mechanical and thermal properties [98]. Furthermore, nowadays, with the presence of conductive polymers like PEDOT: PSS, the compromise in the electrical conductivity is also reduced to a great extent. Although the quality of the MWCNT composite-based strain sensors is better than the pure ones due to their higher selectivity, the pure ones have other advantages like simpler design, avoidance of any kind of distribution of the nano-fillers in the polymer matrix, and chances of agglomeration [99]. 

### 2.1. Pure Multi-Walled Carbon Nanotube (MWCNT)-Based Sensors

Among the earlier research work related to the use of pure MWCNTs for strain sensing, Vemuru et al. [100] explained the analysis related to the real-time response of the prototypes. MWCNT films were developed using a surfactant Nanosperse AQ, where the MWCNTs were sonicated, filtered, and peeled off from the filter to obtain free-standing nanotube films. The films had a density of 50% and a thickness of around 30–50 microns. These sensors were tested for their macroscale responses under tensile load. The responses of the prototypes were also detected in terms of voltage as a function of temperature. A brass specimen was used to attach the MWCNT films to determine the voltage through four-point probe measurements. A linear response of voltage was obtained when the MWCNT films were subjected to tensile strain. The linearity in the responses was maintained until 100 micro-strains, after which the response became non-linear. The films completely recovered in their unstretched state during the unloaded condition. The MWCNT films, when attached, the brass specimen had a Young’s modulus of 166 GPa and a gauge factor (G.F.) of 0.3482. The increase in temperature also led to an increase in the electrical conductivity of the MWCNT films. The resistance values changed by 0.0217 Ω with a temperature difference of 13.9 °C. Another work on the use of standing MWCNT-based films for strain sensing was presented by Zhang et al. [88]. These sensors were operated as resistive devices, where the MWCNT networks were reinforced by PDMS films. The sensors displayed high sensitivity toward both low and high strains. The advantage of these sensors was the customization of the performance of the sensors by adjusting the length and density of the MWCNTs. A gauge factor (GF) of 4.5 was obtained with the highest stretchability of 120%. The sensors were also used for human motions, where the movements of the fingers were monitored to analyze their capabilities for real-time applications. PECVD was used to develop vertical MWCNTs on a thin-film layer of silicon dioxide substrates. Fe was used as a catalyst along with high vacuum conditions. Other experimental conditions included a high temperature of 850 °C, and the presence of H_2_ and N_2_ gases with flow rates of 30 sccm and 10 sccm, respectively. After the MWCNTs were produced, they were transferred on PEN films that had a thin-film layer of PDMS with a thickness of 100 microns. The entire samples were then cured at 70 °C for 15 min, after which the second layer of PDMS was coated with a thickness of 15 microns. Then, the samples containing the MWCNTs and uncured PDMS were covered and cured at 70 °C for an hour. Finally, the silicon substrates on which the PEN films were positioned were peeled off to use the sensors for experimental purposes. 

**Figure 2:**
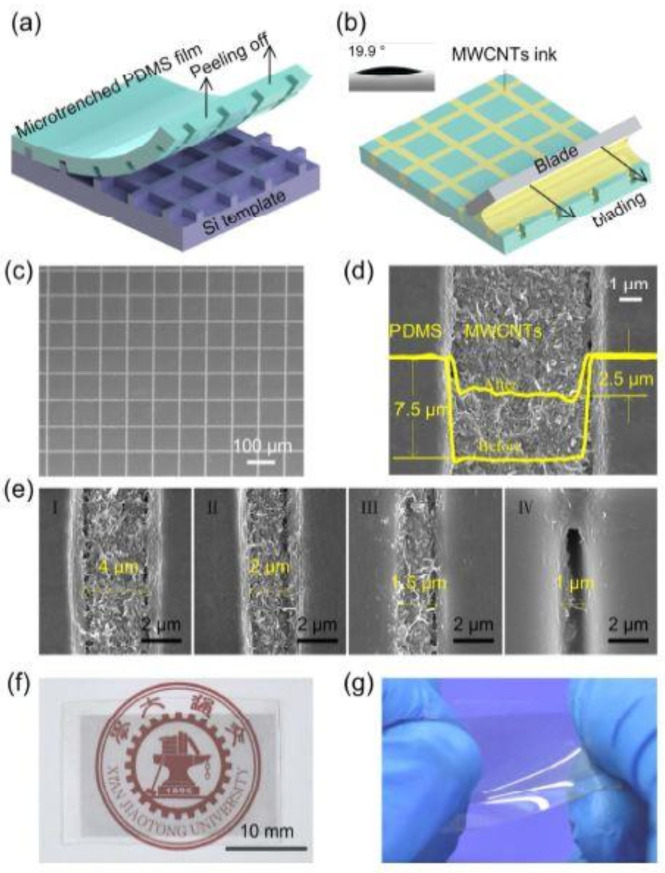
(**a**,**b**) Fabrication process of the multi-walled carbon nanotube (MWCNT)-based sensors formed using flexible PDMS films having mesh-like microtrenches. (**c**) Scanning electron microscope (SEM) images of the developed sensors with MWCNTs embedded in the microtrenches. (**d**) Profiling of the microtrenches before and after the embedding process. The formed sensors were kept in (**e**) normal, (**f**) weisting positions and (**g**) Representation of high stretchability of the sensors. [74]. Reproduced from Nie, B.; Li, X.; Shao, J.; Li, X.; Tian, H.; Wang, D.; Zhang, Q.; Lu, B. Flexible and transparent strain sensors with embedded multiwalled carbon nanotubes meshes. *ACS Appl. Mater. Interfaces* **2017**, *9*, 40681–40689.

Nie et al. [74] showed the use of flexible and transparent strain sensors that were formed using MWCNT meshes. These meshes were embedded in PDMS films for characterization and testing purposes. Mechanical balding of MWCNT-based aqueous dispersions was done into the microtrenches of prestructured PDMS films. Figure 2 [74] depicts the fabrication and profiling steps of these MWCNT-based sensors. The mixture of PDMS was initially drop cast on silicon templates, followed by their desiccation. These silicon templates containing microtrenches were fabricated using conventional photolithography and dry etching techniques. The samples were then cured at 80 °C for 6 h to obtain the microtrenches on the PDMS substrates. Then, oxygen plasma treatment was done on the microtrenches containing PDMS films with a power of 300 W for 50 s. The drop-casting of MWCNTs was done on the micro-trenched PDMS films, followed by filling the nanotubes into the trenches with a doctor blade. The samples were then dried at 60 °C for 5 min and an ethanol-assisted scraping technique was used to remove the excess MWCNTs from the PDMS surfaces. Finally, the samples were sintered at 130 °C for 30 min before using them for characterization and experimental purposes. The sensors showed high optical transparency of 87% and G.F. of 1140. The sensors showed high stability in the responses for over 2000 stretching and release cycles. Along with high sensitivity toward a small strain of 8.75%, the prototypes were also able to respond to body motions like wrist, bending, eye blink, mouth phonation, and pulse.

Sahatiya et al. [89] described the use of MWCNTs to develop flexible, large-area matrixes for strain and pressure-sensing applications. The advantages of these sensors include their eco-friendly and bio-degradable nature. The sensors were formed using pencil eraser substrates using solvent-free, low-cost, and energy-efficient techniques. The reasons for using the erasers were to serve as both substrate and dielectric material for the sensors. The interfacial bonding of the MWCNTs and eraser substrates was increased by a rolling pin and pre-compaction mechanical press. The erasers were initially cleaned with DI water and isopropanol, followed by drying at 70 °C for 20 min. Then, MWCNTs were formed on the substrates using a rolling pin. Different rolling cycles were carried out to ensure the uniformity of the films. The erasers were then dried again at 70 °C for 30 min, followed by cutting them into the desired lengths and widths. The silver conductive paste was used to form the contacts of the sensors. The last step involved the passivation of the eraser substrates with polyimide tapes. The recoverability of the sensors was high in ambient conditions. The strain sensing mechanism was carried out using the tunneling effect of the MWCNTs and strong interlocking between the nanotubes and substrates. The sensors had a G.F. of 2.4 and a sensitivity of 0.135 MPa^−1^. The sensors were connected to different parts of the human body to determine their corresponding movements and touch sensations. 

Huang et al. [68] showcased a similar work where the MWCNT-based sensors were used for strain sensing by operating on vibrational motions. The advantages of these sensors include their high sensitivity, high precision, fast response, and high stability in responses. The MWCNTs were prepared by using a vacuum filtration method, which was subsequently encapsulated with PDMS substrates. Initially, after the rectangular polytetrafluoroethylene (PTFE) molds were formed and attached to glass substrates, PDMS was poured on the molds and heated at 60 °C for 30 min. Then, MWCNTs were deposited on the solidified PDMS layer. Then, the electrodes were formed on the sensors by attaching two copper tapes with silver paste. The silver paste was cured at low temperature to fix the copper tapes at a distance of 20 mm from the MWCNT film. Finally, a second layer was formed on top of the PDMS layer to form a sandwich structure. The second layer was poured on the MWCNT film and subsequently cured in the vacuum oven at 60 °C for an hour. The sensors had a high G.F. of 214.3, which was obtained at a flexural strain of 0.4%. The vibrational motions induced by a base excitation and impact hummer were studied in terms of time and frequency. The responses obtained by the sensors were comparable to an accelerometer with differences of less than 1%. The prototypes also demonstrated high potential as electronic skin sensors when they were tested to detect human motions. 

**Figure 3:**
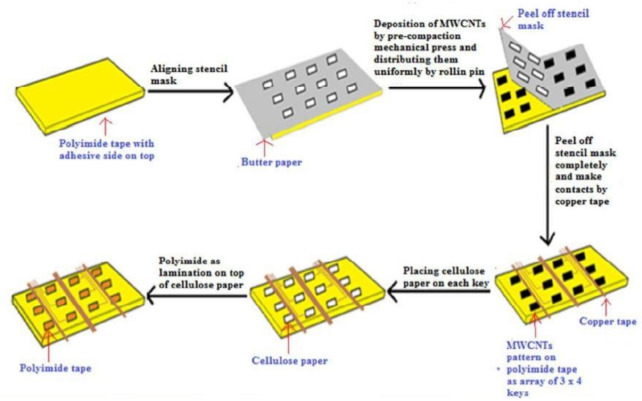
Schematic diagram of the fabrication of the polymide (PI)/cellulose/MWCNT-based strain sensors [101]. Reproduced from Sahatiya, P.; Badhulika, S. Solvent-free fabrication of multi-walled carbon nanotube based flexible pressure sensors for ultra-sensitive touch pad and electronic skin applications. *RSC Adv.* **2016**, *6*, 95836–95845.

Another work [101] showcased the solvent-free fabrication of MWCNTs for developing flexible strain sensors for their use as ultra-sensitive touchpads and electric skins. The advantages of these sensors include their scalability and easy integration to large areas for mapping spatial pressure distribution. Rolling pin and pre-compaction mechanical pressing techniques were used to sandwich the developed MWCNTs between the PI substrate and a protective cellulose paper. Figure 3 [101] shows the schematic diagram of the fabrication process. The adhesive side of the PI tapes was used in the active area during the fabrication process. These substrates were cleaned and used to deposit MWCNTs through an aligned mask. Uniform rolling was ensured by rolling in a single direction. The number of rolling pin cycles and the weight of the MWCNTs were optimized to ensure the uniformity of the MWCNT films. The rolling pin cycles had a range from 50 and 3000 cycles. The decrease in the number of rolling cycles increased the thickness of the MWCNT films. A range of weights were MWCNT deposited on each of the keys, whose values proportionally varied with the non-uniformity of the films. The uniformity of the films was further ensured by performing a pre-compaction mechanical press with a pressure value of 5 kg/cm^2^ for 15 s. Finally, the lamination of the sensors was done using cellulose filter paper on top of the patterned MWCNTs. Then, the mask was taken off to form the MWCNT patterns as touchpads. The contact pads for the measurement of resistive values were formed using silver paste. Pressure, tensile, and compressive forces were measured using the developed sensors. The sensors were employed on artificial skins with 3 × 4-pixel arrays to determine the shape and location of the measured objects. The sensors had a sensitivity, response time, and power consumption of 0.549 kPa^−1^, <32 ms, and <1.9 mW, respectively. 

### 2.2. Composite MWCNT-Based Sensors

The research work on the formation of MWCNT composites has been done extensively for strain sensing applications. The advantages of using MWCNTs instead of pure ones lie in the additional advantages of the processed materials added to the composites. Scientists have tried different mixed kinds of polymers and nanoparticles to enhance the quality of MWCNT nanocomposite-based sensors [102,103,104]. These sensors have proven to have higher sensitivities in terms of G.F., lower response time, and lower hysteresis. The research work highlighted below shows some of the significant examples of the usage of different kinds of nanotechnology-based materials to form MWCNT composites. The developed prototypes were used for monitoring human health in terms of limb movements, physiological parameters, and other associated applications. The nanocomposite-based sensors were categorized into sub-sections based on the type of materials used to form them.

#### 2.2.1. MWCNTs/PDMS Strain Sensors

The use of PDMS to mix with MWCNTs to form nanocomposite-based strain sensors have been the most common in this sector. Researchers have largely mixed MWCNTs with PDMS to exploit the advantages related to nanocomposite-based sensing. PDMS has been preferred as a polymer to its biocompatible and hydrophobic nature, which assists in linking these sensors for the detection of human-related motions. One of the works can be seen in [105], where non-covalent functionalization of MWCNTs was done to develop piezoresistive strain sensors. The sensors were tested for low-pressure regimes with values of less than 100 kPa. The electrodes of the sensors were formed by mixing vinyl-terminated PDMS with poly(phenylmethyl siloxane) and MWCNTs. The functionalization was carried out in two steps, with the first step involving the stacking of low molecular π–π PPMS on MWCNTs, followed by wrapping the MWCNTs using vi-PDMS. The samples were then sonicated for 10 min, after which they were dried at 120 °C for an hour. The homogenous dispersion of the nano-fillers in the polymer matrix was obtained, which helped in increasing the overall conductivity and sensitivity of the prototypes. Then, these functionalized MWCNTs were again ultrasonicated, dried, and taken for the formation of nanocomposites. The functionalized MWCNTs were then mixed with chloroform and PDMS, which were then desiccated and molded on flexible polyamide substrates to form the flexible sensors. Finally, a silver paste was painted and used as electrodes. Prior to the experiments, the samples were cured at 120 °C for 30 min. A percolation threshold of 0.2 vol.% was used to develop these piezoresistive devices. The sensitivity, electrical conductivity, and Young’s modulus of the sensors were 22.16 × 10^−3^ kPa^−1^, 5.43 × 10^−3^ S/m, and 288.83 kPa, respectively. Another interesting work was shown by Li et al. [106], where a simple, facile strategy technique was used to develop flexible piezoelectric sensors based on MWCNTs. The sensors were breathable and washable in nature that contained nanofibers coated with MWCNTs. The sensors were developed in two stages, with the first one involving the coating of the surface of the poly(vinylidene fluoride-co-hexafluoropropylene) (PVDF-HFP) nanofibers via the electrospinning technique. The second step involved the thermal welding of the MWCNTs into nanofibers to prevent the damage of these nanotubes during the washing and experimental processes. The sensors exhibited excellent strain-sensing properties with high stability in the responses up to 10,000 cycles. The nanofiber sheet had a high electrical conductivity with a sheet resistance of 7.1 ± 2.8 kΩ. 

**Figure 4:**
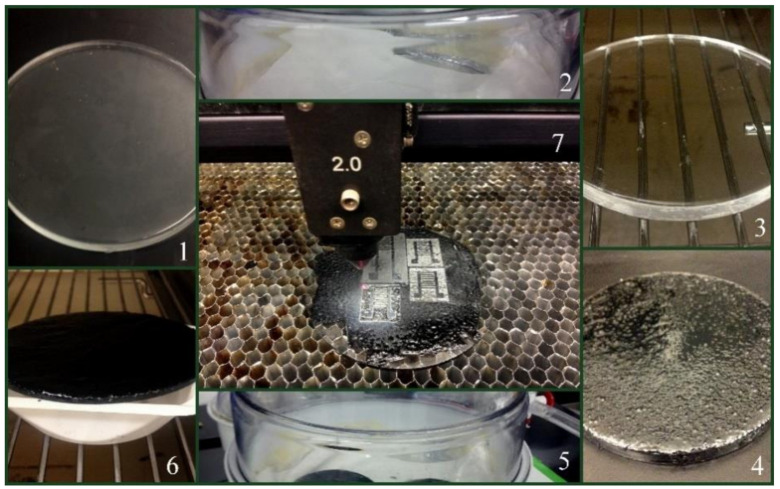
Representation of the steps of fabrication that were followed to develop the MWCNT/PDMS-based strain sensors [107]. Reproduced from Nag, A.; Mukhopadhyay, S.C.; Kosel, J. Flexible carbon nanotube nanocomposite sensor for multiple physiological parameter monitoring. *Sens. Actuators A Phys.* **2016**, *251*, 148–155.

Nag et al. [107] showed the use of MWCNT-based flexible sensors determining limb movements and respiration. The sensors were fabricated using a nanocomposite that comprised of functionalized MWCNTs and PDMS. The amount of MWCNTs was optimized to 4 wt.% to obtain a balance between the electrical conductivity and mechanical flexibility of the prototypes. Figure 4 [107] shows the fabrication steps that were followed to form the prototypes. Initially, a PDMS layer was cast on a polymethyl methacrylate (PMMA) template to form the substrate of the sensors. The thickness of the PDMS layer was adjusted to 1000 microns using a casting knife. After this layer was desiccated and cured at 70 °C for 8 h, a layer of nanocomposite was cast on the top of the PDMS layer. The thickness of this layer was adjusted to around 600 microns. Then, the samples were again cured at the same conditions and taken for developing the electrodes. Laser cutting was employed to form the sensors, where certain parameters like power, speed, and *z*-axis were optimized to obtain proper cuts in the electrodes. The optimized values of these parameters were 24 W, 70 m/min, and 1 mm, respectively. The sensors were tested for the detection of limb movements by attaching them to the joints of the arm and leg. The flexed and extended conditions of the experimental process were analyzed in terms of the change in capacitive values. For the monitoring of respiration, the sensors were attached to the lower part of the diaphragm to detect the inhalation and exhalation through the tensile and compressive movements of the sensor patches. The sensors were capable of detecting both of these applications to validate their functionality for wearable electronics for the detection of strain-induced human motions. 

A very similar work was shown by Giffney et al. [95], where highly stretchable printed strain sensors were developed using similar MWCNTs and silicone rubber nanocomposites. The sensors were employed as piezoresistive large strain sensors that showed high potentiality for wearable electronics and soft robotics applications. The nanocomposites were formed by adding the MWCNTs to the uncured silicon at a defined mass ratio. These samples were then taken for centrifugation to form a uniform dispersion. A trade-off had to be made in the amount of MWCNTs added to the silicone rubber to balance the electrical conductivity and viscosity of the printed materials. Then, the sub-surface printing process inside the partially cured insulating matrix was carried out to achieve conductive traces inside the embedded prototypes. The printing, when carried out in the x–y direction, was done sufficiently deep enough to avoid the cracking of the surface when the prototypes were stretched. The nanocomposites were then taken into syringes under vacuum conditions. The silicone rubber embedding the conductive material provided additional advantages of insulation and protection against wear. The sensors displayed a maximum hysteresis of 11% for a corresponding maximum strain of 300%. The G.F. ranged between 1 and 1.5 for the developed sensors.

One interesting research related to the use of MWCNTs for strain sensing was shown by Momin et al. [108]. This work explained the development of compact load cells using composites that are formed using MWCNTs and cotton. The first step of the fabrication process involved the functionalization of the MWCNTs using citric acid-assisted oxygen plasma. The MWCNTs underwent various processes like sonication, drying, and oxygen plasma treatment. The functionalized MWCNTs were then dispersed in water, in which natural cotton aggregates were dipped and dried several times at room temperature. The load cells were generated using a holder made of a plastic housing case with specific dimensions. The parallel electrodes of the load cell were formed using nickel sheets on which two thin copper wires with a diameter of 0.3 mm were attached. The sensors were used for monitoring human health and activities. The sensors were attached to the center of the human foot to determine the changes in the center of gravity for sick and healthy people. The sensitivity of the sensors ranged between 8.84 Pa and 884 kPa for a tested force ranging between 1 mN and 100 N. A rapid response of 4.5 and 5 ms was obtained for the application and release of the load, respectively. The sensors demonstrated high reproducibility in their responses for the compression/relaxation done over 11,200 cycles. Other qualities of the sensors involved their good electrical conductance recovery property and capability of working in harsh environments. 

#### 2.2.2. MWCNT/Polyurethane-Based Strain Sensors

The presence of polyurethane in the strain sensors enhances the quality of the prototypes in terms of abrasion and tear resistance. They also have high impact resistance and excellent wear properties. He et al. [109] showed the fabrication and implementation of highly stable and flexible sensors using composites formed with modified-MWCNTs and polyurethane (PU) films. The surface modification of the MWCNTs assisted in the improvement of their dispersibility and compatibility in the polymer matrix. The modification was done using silane coupling agent KH550 and dodecylbenzene sulfonate (SDBS). A total of 1 wt.% of KH550 and SDBS were mixed in ethanol and MWCNTs. Sonication, centrifugation, and drying of these solutions were undertaken to form the modified-MWCNTs. These MWCNTs were then mixed with *N*,*N*-dimethylformamide and PU resin to form the composites. The homogenous solutions were then ultrasonicated and cast on the glass mold. These molds were then dried in a vacuum oven at 85 °C for 3 h to form the sensors. The dimensions of the final prototypes included a surface area of 28 cm^2^ and a thickness of 50 microns. The sensors were employed for different strain-induced motions like respiration, and the human pulse of the neck and wrist. The sensitivity of these sensors was 4.282% kPa^−1^ for a pressure ranging within 1 kPa. The electrical conductivity increased with the increase in frequency for values over 10 kHz. The errors related to the repeatability, hysteresis, and non-linearity were 54.95%, 16.72%, and 9%, respectively.

A similar work was shown by Kumar et al. [96], where the development and utilization of strong, stretchable, and ultrasensitive piezoresistive sensors were done for the detection of small and large strain regimes. The sensors were formed using nanocomposites that had thermoplastic polyurethane (TPU) and MWCNTs at defined proportions. The nanocomposite films were formed by the solution mixing technique where optimization was done on TPU and MWCNTs by mixing these two components at different concentrations. This was carried out by mixing TPU with DMF, followed by mixing dispersed MWCNT solutions into the former TPU solutions. Finally, the MWCNT/TPU nanocomposites were heated in the oven at 80 °C for 12 h to form the piezoresistive samples. A low percolation threshold and superior electrical conductivity were obtained for the TPU-reinforced MWCNT-based nanocomposites. For a MWCNT loading of 1 wt.% on TPU nanocomposites, the G.F. of the sensors for a strain of 15%, 35%, and 45% were 22, 8.3, and 7, respectively. However, by varying the MWCNT loading by 0.3, 0.5, and 1 wt.%, the G.F. values changed to 6395, 6423, and 7935 for strain values of 35%, 95%, and 185%, respectively. The tensile strength, yield strength, and Young’s modulus of the nanocomposites increased for MWCNT loadings of 0.1 wt.% and 0.3 wt.%. The sensors also showed high recoverability and reproducibility of the results for stretch and release tests for over 100 cycles for a strain-amplitude of 50%. 

Another work in the use of PU can be shown in the research done by Wang et al. [110], where high stretchable strain sensors were formed using MWCNTs and TPU fibers. The TPU fibers containing porous structures were mixed with MWCNTs using a simple and cost-efficient wet-spun method. The primary step in this technique involved the solvent exchange during the solidification process, which assisted in the improvement in the sensing range. The MWCNTs with a diameter of around 8–15 nm, length of 20–50 microns, and specific surface area of 250–300 m^2^/g were considered for forming the nanocomposites. Prior to their use, these nanotubes were dried at 120 °C in a vacuum for 3 h. The MWCNTs were mixed with DMF to form homogenous solutions. Then, TPU was used to form suspensions, which were mixed with the MWCNT-based solution. Then, these samples were sonicated and filtered using DI water to obtain the final nanocomposites. These composite fibers were cut into 5 cm to form the sensing area of the prototypes. Finally, two copper tapes were attached to the two ends of the composite fibers using a silver paste to form the electrodes of the sensors. The sensors displayed an ultra-wide workable strain range of 320%, with a fast response time of 320 ms. The G.F. of the sensors were 22.2 and 97.1 for the range of strain of less than 160% and 160–320%, respectively. The prototypes also exhibited high reproducibility and excellent durability in their responses during the multi-cycle testing conducted over 9700 cycles at 100% strain. The sensors were beneficial for human-related strain-induced motions like the bending of fingers, elbow, knee, and other activities like squatting and squat-jumping. 

**Figure 5:**
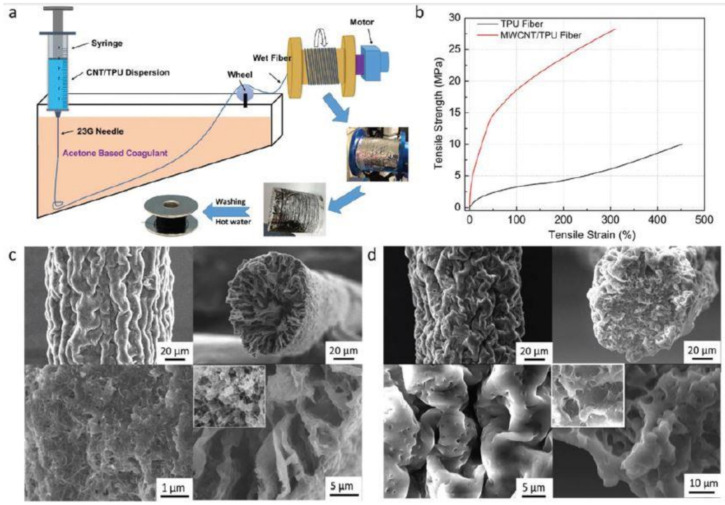
(**a**) Schematic diagram of the wet spinning process of the MWCNT-based composites. (**b**) Stress–strain curve of the composites. (**c**,**d**) SEM images of the composite fibers [91]. Reproduced from He, Z.; Zhou, G.; Byun, J.H.; Lee, S.K.; Um, M.K.; Park, B.; Kim, T.; Lee, S.B.; Chou, T.W. Highly stretchable multi-walled carbon nanotube/thermoplastic polyurethane composite fibers for ultrasensitive, wearable strain sensors. *Nanoscale* **2019**, *11*, 5884–5890.

He et al. [91] explained a similar work related to the fabrication of wearable strain sensors using composites developed from MWCNTs and TPU fibers. The wet spinning process was used to generate ultrasensitive prototypes with high tensile strength. Figure 5a [91] shows the schematic diagram of the fabrication process. The diameters of the nanocomposites of MWCNT/TPU and pure TPU fibers were 130 microns and 110 microns, respectively. The suspensions created by mixing MWCNTs and TPU were extruded into a coagulation bath, from which the wet fibers were collected. The fibers were then treated with hot water to remove the surfactants attached to the composite fibers. The fibers were then collected on thread pools for sewing purposes. The sensors containing TPU fibers with a weight ratio of 1:8 had a tensile strength of 28 MPa and a failure strain of 310%. The G.F. of the sensors was around 2800 for a strain ranging between 5 and 100%. The sensors were used for determining the weights and shapes of different objects based on the 2D mapping of resistive changes. The prototypes were also employed as wearable sensors by stitching them onto stretchable elastic bandages using a sewing machine. These sensing systems were used for determining diverse human motions by using it as a smart glove to detect the movement of the fingers. PU has also been used in other forms during its association with MWCNTs to form strain sensors. PU has also been used in other ways, as shown by Hong et al. [111]. Skin-like stretchable arrays of multifunctional sensors were formed using MWCNTs and polyaniline nanocomposites. The facile method was used to develop stretchable sensors that were used as electronic skins and human monitoring systems. PU was used as a foam to coat the sensors, which were used for the detection of body temperature, wrist pulse, and ammonia gas. The sensors displayed stable performances under a strain limit of 50%. 

#### 2.2.3. MWCNT/Silver Nanoparticle-Based Strain Sensors

**Figure 6:**
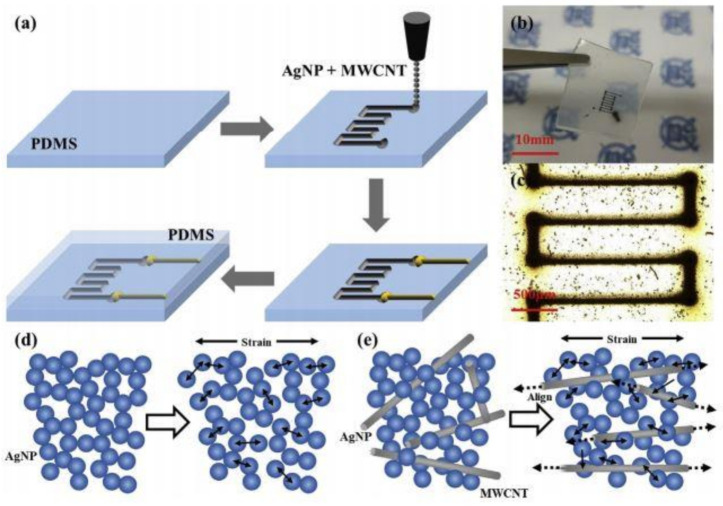
(**a**) Schematic diagram of the fabrication steps of the Ag NP/MWCNT-based sensors. (**b**) Photograph and (**c**) optical images of the fabricated samples. (**d**) Schematic diagram of the operating principle of the nanocomposite-based strain sensors and (**e**) Movement of the MWCNTs and silver nanopartciles under stretched condition. [112]. Reproduced from Min, S.H.; Lee, G.Y.; Ahn, S.H. Direct printing of highly sensitive, stretchable, and durable strain sensor based on silver nanoparticle/multi-walled carbon nanotube composites. *Compos. Part B Eng.* **2019**, *161*, 395–401.

MWCNTs have also been mixed with silver nanoparticles to increase the sensitivity of the prototypes. The high aspect ratio and enhanced electromechanical properties of the silver nanoparticles have assisted researchers in generating high-quality sensors. The authors in [112] showed the development of high-quality sensing prototypes via direct printing using aerodynamically focused nanoparticle printing systems. The printing of dry and inorganic silver nanoparticles (Ag NPs) was done through direct deposition in a low vacuum and room temperature. The printed materials comprised microscaled porous conductive patterns of Ag NPs and MWCNTs. The design of the electrodes was printed on PDMS substrates to obtain highly sensitive, stretchable, and durable strain sensors. Figure 6a [112] shows the schematic illustration of the fabrication process of the Ag NP/MWCNT-based sensors. EcoFlex 0030 was used as substrates to form the sensors. AFN printing was carried out to form the designated electrode designs on the templates. This layer was wired and soldered for measurement purposes. The silver paste was used to connect the tip of the prototypes to the electric wires. The images of the developed sensors are shown in Figure 6b,c [112]. The sensors had a length of 7 mm, a width of 3.5 mm, and a distance between patterns of 0.5 mm. The operating principle of these sensors was based on the degree of attachment/detachment of the Ag NPs with the MWCNTs for the respective tensile and compressive strains. The response of the sensors was studied in terms of the relative resistance with respect to the applied strain. Then, another flexible layer of PDMS was formed on top of the prototypes for electrical insulation and mechanical protection. The sensors projected high mechanical stability toward the tested range of strain values. A G.F. of 58.7 was obtained for a strain limit of 74%. The variation in the responses was less than 5% for the tests conducted over 1000 loading–unloading life cycles.

**Figure 7:**
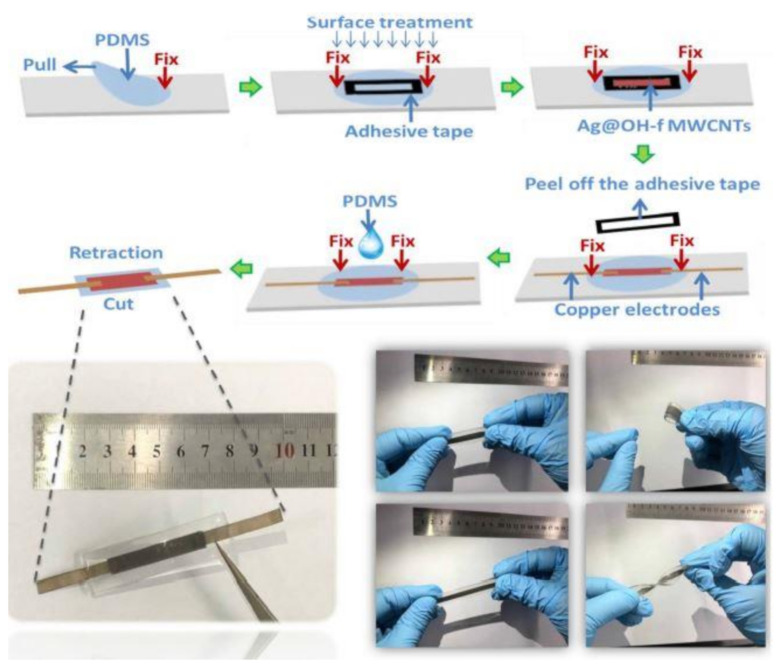
Flow of the fabrication process of the Ag NP/MWCNT-based strain sensors [113]. Reproduced from Yuan, Z.; Pei, Z.; Shahbaz, M.; Zhang, Q.; Zhuo, K.; Zhao, C.; Zhang, W.; Ma, X.; Sang, S. Wrinkle Structured Network of Silver-Coated Carbon Nanotubes for Wearable Sensors. *Nanoscale Res. Lett.* **2019**, *14*, 1–8.

Another work on the use of Ag NPs for developing strain sensors can be seen in Yuan et al. [113]. Wrinkled structured networks of Ag NPs were mixed with a hydroxyl group (OH)-functionalized MWCNTs to form sensors for highly acute sensing. Deposition methods were used to fabricate the sensors, which had certain features like flexibility, ultra-lightweight, and high comfortability as wearable prototypes. Figure 7 [113] shows the flowchart of the fabrication process of the sensors. A simple reduction method was used to develop the sensors. The functionalized MWCNTs were mixed with silver nitrate aqueous solutions and subsequently heated in an oil bath. Then, sodium citrate was added to the mixture along with heating and stirring it. Then, PDMS substrates were taken and stretched to 110% by attaching them with fixed adhesive tapes. A rectangular hole was formed on the substrates, followed by treating the surface with Schwarze P3C for 300 s. The MWCNT-based composites were dropped on the rectangular hole of the stretched PDMS film. The tapes were then removed, followed by the pasting of two copper electrodes on top of the electrodes. In order to further strengthen the connection between the nanocomposites and electrodes, PDMS solutions were dropped on top of the electrodes and heated at 75 °C for an hour. The G.F. of the sensors was 412.32 for a strain of 42.2%. The sensors were used for the detection of physiological movements like facial and hand movements. The devices were also used for monitoring behavioral features like the breathing of an individual.

A similar work conducted recently can be seen in [114], where the authors used a facile strategy to develop highly stretchable strain sensors for monitoring human movements and human–robotic interactive systems. The sensors were fabricated using the micro molding-in-capillary process, where silicon rubber was used to wrap the sensing material to form a sandwich-like structure. The conductive part of the sensors was made up of nanocomposites formed using MWCNTs and silver nanowires. The sensors exhibited a wide sensing range of 2% to 180%, along with high durability to the applied strain. The MWCNTs present in the composites had responses toward strain ranging between 45 and 120%. The G.F. of the sensors was over 10^6^ for a strain of 30%. The stability in the responses was high for over-tested 6000 cycles. The sensors were tested for monitoring the motions of different body parts like the finger, knee, and wrist. The sensors were also capable of responding to multichannel and interactive electronic systems by assisting in the control mechanism for the teleoperation for robotic end-effectors.

## 3. Future Scope of MWCNT-Based Strain Sensors

Even though a lot of work has been done related to the use of MWCNTs for strain-sensing applications, there are still some bottlenecks that need to be addressed to improve the quality of the prototypes. The above-mentioned research work related to the use of MWCNTs to combine with different kinds of polymers and conductive nanomaterials formed highly efficient strain sensors that showed enhanced performance in terms of G.F., stretchability, and detection range. 

The use of different materials to compose the above-mentioned pure and composite strain sensors was based on the individualistic pros and cons associated with them. The inclusion of each of these materials has helped in enhancing the performance of the strain sensors. Certain processing materials like PDMS have been used to form both the substrates and composites of the sensors and has assisted in building robust sensors through the proper attachment of the electrodes. In addition to being biocompatible and hydrophobic in nature, it has allowed research groups to customize its mechanical flexibility and thickness to form the substrates of the sensors by optimizing the ratio between the pre-polymer and curing agent. Other advantages of PDMS include low cost and optical transparency, which not only decreases the overall fabrication cost but also increases the dynamicity in terms of applications of the prototypes. Another major advantage of PDMS for strain sensors is the hydrophobic nature it provides during wearable sensing applications. This property ensures the minimal disturbance of the body fluids on the responses during the experimental process. Other materials processed to form the substrates of the pure MWCNTs strain sensors include silicon and PEN, where the former one provides numerous advantages like small size, low hysteresis, the capability to operate in extreme conditions, and high repeatability in terms of large-scale production. The silicon-based sensors, as shown above, also give insights to researchers to integrate carbon-based and metallic nanomaterials with semiconducting elements. One of the issues with the use of silicon is the high cost related to its processing from single-crystal silicon with a thin oxidized layer above it. Certain techniques like 3D printing that contain PTFE molds to form the MWCNT-based strain sensors also had pros and cons associated with them. The advantages of PTFE molds include excellent release, reduced abrasion, and reduced build-up, while the disadvantages are high matrix impurity rate and high cost. 

In order to form the composites of MWCNT-based strain sensors, PU and silver nanoparticles are additional materials that are used other than PDMS. PU is one of the popular materials used to form strain sensors due to its high tear resistance and high tensile properties. They also show strong bonding properties while used in nanocomposites, along with enhanced performance in mild and harsh environmental conditions [115]. One of the major disadvantages of PU includes its low life expectancy, which would require the prototypes to be changed at frequent intervals. This would increase the overall cost of the sensing systems. Another disadvantage of PU is the water absorption characteristics, which might affect the output of the PU-based prototypes when used as wearable sensors. The inclusion of silver nanoparticles in the nanocomposites provided certain advantages like enhanced electrical properties as a result of increased conduction paths, increased stability, and lower detection limits. Toxicity is one of the issues with silver nanoparticles, but this is mostly related to the prototypes that are used for biosensing applications. 

There are still some steps that can be taken to ensure further quality control of the MWCNT-based prototypes. Since the mixing of MWCNTs with aqueous solutions without the presence of any surfactants is still an issue, further functionalization should be done on these carbon allotropes, which will assist in their dissolution. Different kinds of functional groups should be added using nucleophilic addition reactions to add groups that would be multivariate in nature. The impurities present during the fabrication of MWCNTs using different techniques like chemical vapor deposition [116,117], arc-discharge [118,119] and liquid electrolysis [120,121] should also be reduced on the group level. This would not only help to improve the grade value of the MWCNTs, but also overall improve the quality of the resultant prototypes. The removal of the generated impurities can be addressed by focusing on the experimental parameters like the temperature of the chamber, catalyst, and gases. The conjugation of MWCNTs with other efficient materials like graphene should be further researched and worked on [122,123]. Due to the excellent characteristics of graphene, they should be intertwined with MWCNTs to introduce additive features to the electrodes. These conjugates would also help in improving the quality of nanocomposites that would have other polymers present in them. While the MWCNTs will assist with the interfacial bonding inside the polymer matrix, graphene would help in the electromechanical properties of the conductive fillers. Another improvement can be made on the alteration of the testing scenario of the fabricated MWCNT-based strain sensors. Although these sensors show excellent performances in laboratories with controlled ambiance conditions, their ubiquitous operation in real-time application is something that will have long-lasting influences on the industrial sectors. These strain sensors should be embedded with other signal-conditioning circuits to perform wireless operation. Once these above-mentioned issues are addressed, the dynamic range of the utilization of MWCNTs for strain and other similar applications would increase to a great extent. While using CNTs and other carbon-based allotropes to fabricate strain sensors, a reduced amount of toxicity of these nanomaterials is one of the parameters that need to be taken into account. This is primarily to ensure that safe operational procedures are being followed during the fabrication process. CNTs are said to have carcinogenic effects that can cause disastrous effects like tumors, pulmonary inflammation, fibrosis, and granuloma in the lungs [124]. However, the intensity of these effects varies depending on the diversified properties of CNTs such as length, aspect ratio, surface area, degree of aggregation, purity, and concentration [125]. Thus, a thorough study using simulations should be performed before using the CNTs and related-allotropes in order to optimize the critical parameters related to them. Additionally, the fabrication process should be carried out in closed chambers in specialized clean rooms to minimize exposure. Each of the fabrication steps carried out to design and develop the sensors followed standardized protocols that have been previously tested and verified. The laboratories containing these nanomaterials should also be equipped with paramedics who can be immediately consulted in the case of an emergency. 

The market scope related to the use of CNTs for strain-sensing and other applications is said to increase exponentially in the near future [126]. There is an estimated rise in the use of CNTs from 4.55 billion USD in 2018 to 9.84 by 2023, with a compound annual growth rate of 16.70% [127]. This considers the fabrication of both SWCNTs and MWCNTs, along with their applications in the electronics, chemistry, energy, and medical fields. The increase is said to be worldwide, especially in countries like India, China, Brazil, and the Middle East due to their emerging economies [128]. The use of MWCNTs in the past few years is said to be more than that of SWCNTs due to their higher tensile strength, a result of which there has been a higher number of applications that try to employ MWCNTs for detection purposes. 

## 4. Conclusions

The paper depicts the use of MWCNTs for strain-sensing applications. These conductive carbon allotropes have been used in pure and composite forms to form prototypes that are capable of detecting a wide range of strains. The parameters related to the strain sensors depend mainly on the structure and processing materials of these sensors. The advantages of these sensors are their low-cost fabrication process, easy operating principle, high robustness, and high stability and repeatability of the responses. The MWCNT-based sensors showed a better sensitivity toward strain sensing for the composite forms compared to the pure ones. These devices have been used for biomedical and industrial applications, where their presence in the form of sensorial systems exerts a great impact on the sensing world.

## Figures and Tables

**Table 1:** Comparative study of some of the significant works done on the use of multi-walled carbon nanotubes (MWCNTs) for strain-sensing applications.

Processed Materials	Limit of Detection	Stretchability	Gauge Factor	Ref.
MWCNTs, PDMS	5%	10%	513	[87]
MWCNTs, PDMS	10%	120%	4.5	[88]
MWCNTs, Eraser	30%	30%	2.4	[89]
MWCNTs, PDMS		146%	12.15	[90]
MWCNTs, Thermoplastic Polyurethane (TPU)	5%	100%	2800	[91]
MWCNTs, Graphite films	5%	100–620%	43.4	[92]
MWCNTs, Silicone rubber	10%	10–40%	34.38	[93]
MWCNTs, Graphene platelets	1%	237.5%	181.36	[94]
MWCNTs, Silicone polymer	1%	300%	1–1.5	[95]
MWCNTs, Thermoplastic Polyurethane (TPU)	0.3%	35–185%	22	[96]

## Data Availability

Not applicable.
